# Properties of Plasmodium falciparum with a Deleted Apicoplast DNA Gyrase

**DOI:** 10.1128/AAC.00586-21

**Published:** 2021-08-17

**Authors:** SooNee Tan, Devaraja G. Mudeppa, Sreekanth Kokkonda, John White, Rapatbhorn Patrapuvich, Pradipsinh K. Rathod

**Affiliations:** a Department of Chemistry, University of Washington, Seattle, Washington, USA; b Drug Research Unit for Malaria, Faculty of Tropical Medicine, Mahidol University, Bangkok, Thailand

**Keywords:** apicoplast, CRISPR/Cas9, ciprofloxacin, DNA gyrase, *Plasmodium falciparum*

## Abstract

Malaria parasites have three genomes: a nuclear genome, a mitochondrial genome, and an apicoplast genome. Since the apicoplast is a plastid organelle of prokaryotic origin and has no counterpart in the human host, it can be a source of novel targets for antimalarials. Plasmodium falciparum DNA gyrase (*Pf*Gyr) A and B subunits both have apicoplast-targeting signals. First, to test the predicted localization of this enzyme in the apicoplast and the breadth of its function at the subcellular level, nuclear-encoded *Pf*GyrA was disrupted using CRISPR/Cas9 gene editing. Isopentenyl pyrophosphate (IPP) is known to rescue parasites from apicoplast inhibitors. Indeed, successful growth and characterization of *Pf*ΔGyrA was possible in the presence of IPP. *Pf*GyrA disruption was accompanied by loss of plastid acyl-carrier protein (ACP) immunofluorescence and the plastid genome. Second, ciprofloxacin, an antibacterial gyrase inhibitor, has been used for malaria prophylaxis, but there is a need for a more detailed description of the mode of action of ciprofloxacin in malaria parasites. As predicted, *Pf*ΔGyrA clone supplemented with IPP was less sensitive to ciprofloxacin but not to the nuclear topoisomerase inhibitor etoposide. At high concentrations, however, ciprofloxacin continued to inhibit IPP-rescued *Pf*ΔGyrA, possibly suggesting that ciprofloxacin may have an additional nonapicoplast target in P. falciparum. Overall, we confirm that *Pf*GyrA is an apicoplast enzyme in the malaria parasite, essential for blood-stage parasites, and a possible target of ciprofloxacin but perhaps not the only target.

## INTRODUCTION

Malaria is a global health problem, and continual emergence of resistance to existing antimalarial drugs underscores the importance of finding new drug targets and new antimalarials (1–6). There are three genomes in the malaria parasites: the nuclear genome, the mitochondrial genome, and the apicoplast genome (7–9). During cell division, DNA topoisomerases play a major role in DNA replication, DNA transcription, and DNA repair throughout the life cycle of parasites (10–13). There are two types of DNA topoisomerases (type I and type II) that are categorized based on their ability to break single-stranded or double-stranded DNA, respectively (10). Malaria parasites have two type I (topoisomerase I and III) and three type II (topoisomerase II and VI and DNA gyrase) enzymes (7). Biochemical and cellular functions of the majority of malarial topoisomerases remain uncertain, partly due to the difficulty in expressing active malaria enzymes. Previously, we successfully expressed full-length topoisomerase II and tested its sensitivity to some known enzyme and parasite proliferation inhibitors (14). Meanwhile, Chalapareddy et al. suggested that topoisomerase VI may be important for maintenance of the mitochondrial genome (15). Based on bioinformatics, DNA gyrase is known to carry an apicoplast-targeting signal peptide (16–18). Although heterologous expression of Plasmodium falciparum DNA gyrase A (*Pf*GyrA) subunit was not successful, Dar et al. showed successful expression of *Pf*GyrB subunit and its localization to the apicoplast (19). The functions of truncated forms of *Pf*GyrA have been studied extensively *in vitro* in several independent studies (20–23). However, the essentiality, biological function, and sensitivity to inhibitors of *Pf*GyrA have not been formally established for blood-stage malaria parasites.

**FIG 1:**
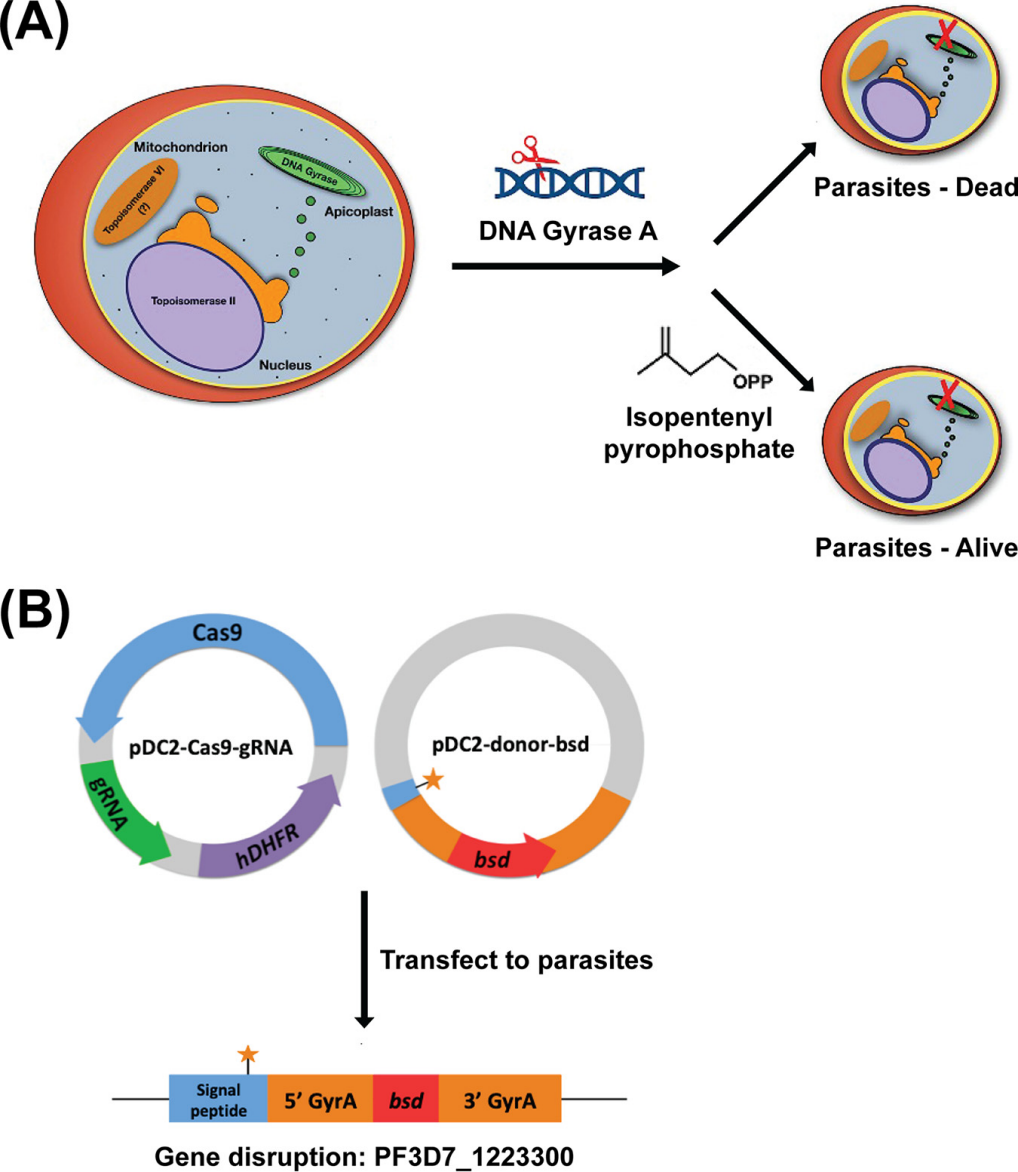
Experimental model and design. (A) *Pf*GyrA was hypothesized to be a type II topoisomerase located in the parasite apicoplast. Disruption of this enzyme was expected to kill parasites if its function is important to the maintenance of the apicoplast. However, genetically modified parasite without an apicoplast can be chemically rescued through supplementation with isopentenyl pyrophosphate. (B) Gene editing with CRISPR/Cas9. The Cas9 enzyme was encoded on a plasmid that also expresses gRNA and an *hdfhr* selectable marker. The donor plasmid carries the homology arms of the *PfgyrA* gene with a point mutation inserted on the 3′ end of the apicoplast-signal peptide. Transfected parasites would have the *PfgyrA* gene disrupted with the insertion of *bsd*.

DNA gyrase is the only type II topoisomerase predicted to localize to the apicoplast and has no counterpart in the human host (17, 24, 25). Since Yeh and DeRisi showed that parasites lacking apicoplast functions can be chemically rescued with isopentenyl pyrophosphate (IPP) supplementation, we hypothesized that deletion of *Pf*GyrA may be tolerated with the addition of IPP (Fig. 1A) (26). If successful, this would formally validate the importance of *Pf*GyrA in malaria parasites and help us understand the mode of action of potential drugs that may target the apicoplast *Pf*GyrA. CRISPR/Cas9 gene editing has been adapted for genome editing in Plasmodium falciparum (27–30). Here, we apply this gene-editing tool to demonstrate that *Pf*GyrA is targeted to the apicoplast, that it is important for the function of the apicoplast, and that it is the primary, but perhaps not the sole, target of ciprofloxacin at high concentrations.

## RESULTS

### CRISPR/Cas9-mediated gene knockout of *Pf*GyrA.

**FIG 2:**
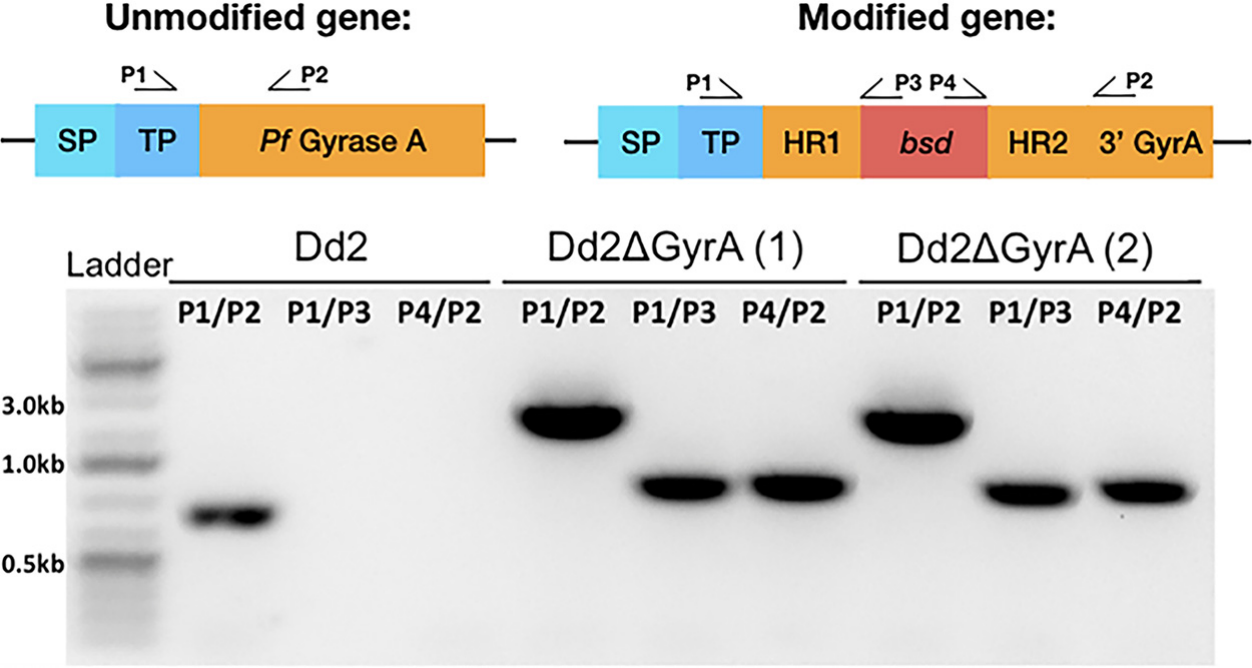
Proof of *PfgyrA* disruption. PCR with different primers helped validate disruption of the *PfgyrA* gene. Genetically modified parasites yielded a larger PCR fragment when amplified with primers P1/P2. Amplification with primers P1/P3 and P4/P2 further confirmed the insertion of the *bsd* gene in the modified parasites. SP, signal peptide; TP, transit peptide; HR, homologous region.

A plasmid, labeled pDC2-gRNA-Cas9, coexpressed Cas9 nuclease and human dihydrofolate reductase (encoded by *hdhfr*) and was selectable in malaria parasites with the antifolate WR99210 (Fig. 1A and B). The donor plasmid, labeled pDC2-donor-bsd, provided a repair template to leverage the homology-directed repair pathway of CRISPR/Cas9. The donor plasmid had homology regions from the targeted *PfgyrA* gene sequence, flanking the blasticidin-S-deaminase (BSD) selectable marker (Fig. 1B). The gRNA-Cas9 and donor plasmids were electroporated simultaneously into a parental P. falciparum Dd2 clone using the direct electroporation method (31, 32). At 48 h posttransfection, parasites were supplemented with IPP and maintained with WR99210 and blasticidin for 6 days, followed by culturing in IPP-supplemented medium. Parasites were visible microscopically on days 21 to 24 posttransfection in most flasks when IPP was supplemented in each culture. Genomic DNA was extracted from each positive transfected clone for downstream confirmation assays. PCR-based *PfgyrA* gene amplifications were performed on each transfected clone using different primer sets to confirm the disruption of *PfgyrA* gene (Fig. 2). Amplification with primers P1/P2 yielded an 898-bp fragment seen in the Dd2 clone, while amplification with the same primer set yielded a larger 2,490-bp fragment from the modified clones (Dd2ΔGyrA). Since the Dd2 clone did not include the *bsd* gene, no fragment was observed when the DNA was amplified with primers P1/P3 or P4/P2. On the other hand, amplification with primers P1/P3 or P4/P2 yielded a 1,110-bp or 1,102-bp fragment in the modified clones and hence confirmed the insertion of the selectable marker. Two out of 13 transfected clones carried the desired modification based on the PCR confirmation. DNA sequencing of the amplified PCR products further confirmed the disruption of the *PfgyrA* gene.

### Loss of apicoplast in Dd2ΔGyrA.

**FIG 3:**
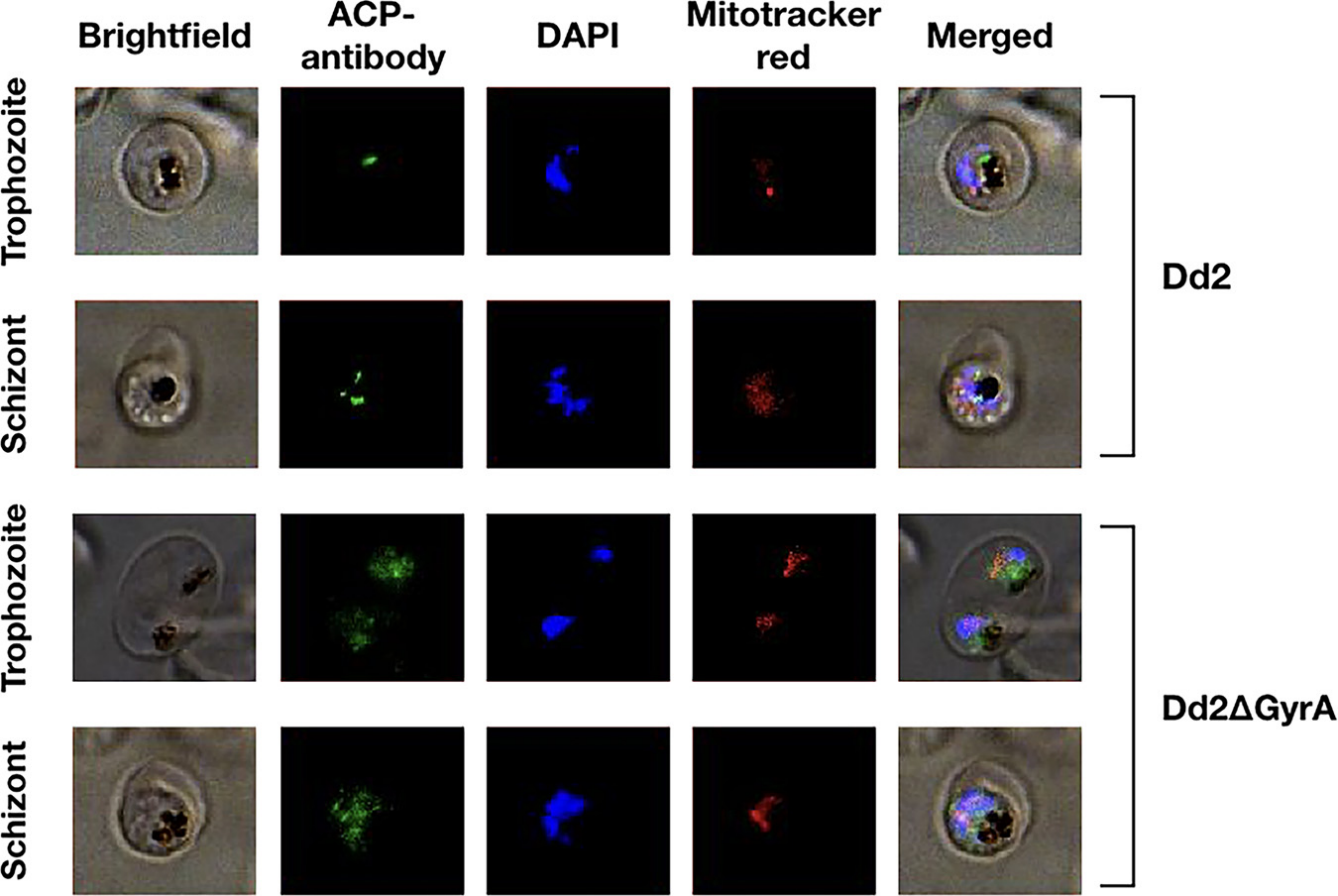
Loss of apicoplast immunofluorescence in Dd2ΔGyrA clone. The parasite apicoplast was stained with the ACP antibody, which was visualized with the anti-rabbit secondary antibody conjugated to Alexa Fluor 488. Nucleus was stained with DAPI, while mitochondrion was stained with the Mitotracker CMXRos. The apicoplast remained intact in the Dd2 clone, as ACPs were localized to a specific location. However, in the Dd2ΔGyrA clone, ACPs were dispersed throughout the cytoplasm of parasites, indicating loss of the apicoplast structure. Nuclear and mitochondrial genome remained intact in both Dd2 and Dd2ΔGyrA clones.

In order to determine whether the apicoplast remained intact after the disruption of the *PfgyrA* gene and growth in the presence of IPP, genetically pure populations of Dd2ΔGyrA were generated through limiting dilution. Apicoplast structure was visualized through staining of an apicoplast-targeted acyl-carrier protein (ACP) with an ACP antibody (33). As expected, the ACP localized to a discrete structure (the apicoplast) in the Dd2 clone, while ACP was diffused in the cytoplasm of the Dd2ΔGyrA clone (Fig. 3). As a control, the nucleus was stained with DAPI (4′,6-diamidino-2-phenylindole) and the mitochondrion was stained with Mitotracker CMXRos. Under these conditions, there was no difference in the staining pattern between Dd2 and Dd2ΔGyrA clones, suggesting that the nucleus and mitochondrion remained intact in these parasites.

**FIG 4:**
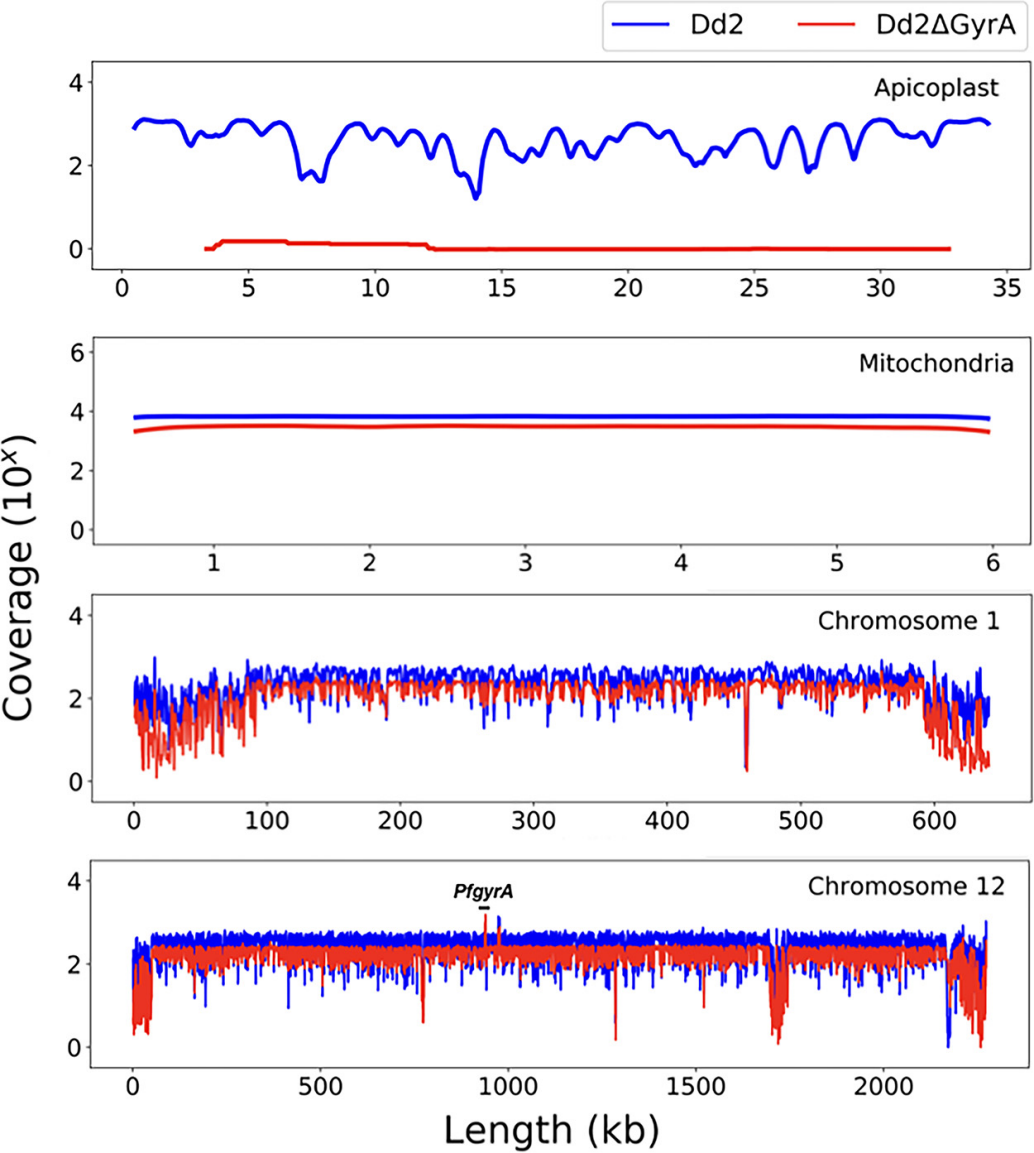
Loss of the apicoplast genome in Dd2ΔGyrA clone. The genome sequence coverage plot (in red) confirmed loss of the apicoplast genome in Dd2ΔGyrA while still retaining the mitochondrial and nuclear genomes. The unmodified Dd2 clone has its apicoplast, mitochondrial, and nuclear genomes intact (in blue).

The loss of the apicoplast in Dd2ΔGyrA was further confirmed through whole-genome sequencing. The coverage of sequences from the whole genome of Dd2ΔGyrA was compared to that of a Dd2 clone. As shown in Fig. 4, Dd2ΔGyrA had lost its apicoplast DNA compared to the parental Dd2 clone. In contrast, as represented by chromosome 1 reads, both mitochondrial and nuclear genomes remained intact in both Dd2 and in Dd2ΔGyrA clones.

### Dependence of Dd2ΔGyrA on IPP for growth.

**FIG 5:**
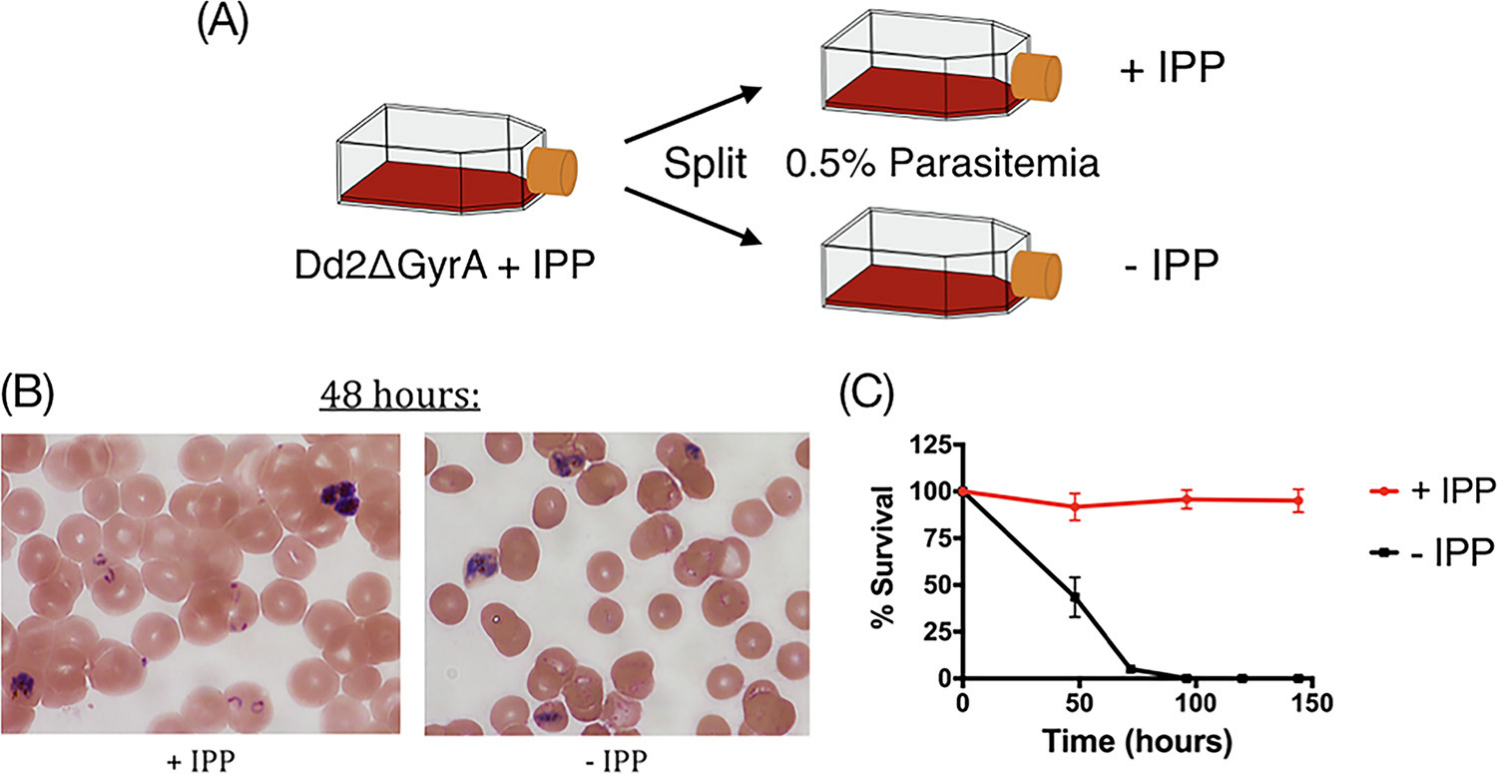
Dependence of Dd2ΔGyrA on IPP for growth. (A) Dd2ΔGyrA clone was split into two independent flasks: one received continuous supplementation of 200 μM IPP, while the other flask received no IPP. (B and C) Parasites with continuous IPP supplementation were able to proliferate and appeared healthy. Parasites in flask without IPP had no healthy rings and were not viable after 48 h.

In order to directly test whether Dd2ΔGyrA clone was dependent on IPP for growth, each flask was split into two. One flask was maintained in the presence of IPP, while the other flask had IPP removed. Parasites were observed microscopically (Fig. 5A). About 48 h after IPP removal, the parasite cytoplasm was less condensed and the morphology of parasites was distorted compared to that of parasites cultured in IPP-supplemented medium (Fig. 5B). While parasites proliferated normally in cultures supplemented with IPP, reduced parasite growth was observed in cultures at 48 h post IPP removal, and parasites were considered nonviable at 72 h post IPP removal (Fig. 5C).

### IPP rescues parasites from delayed-death phenotype.

**FIG 6:**
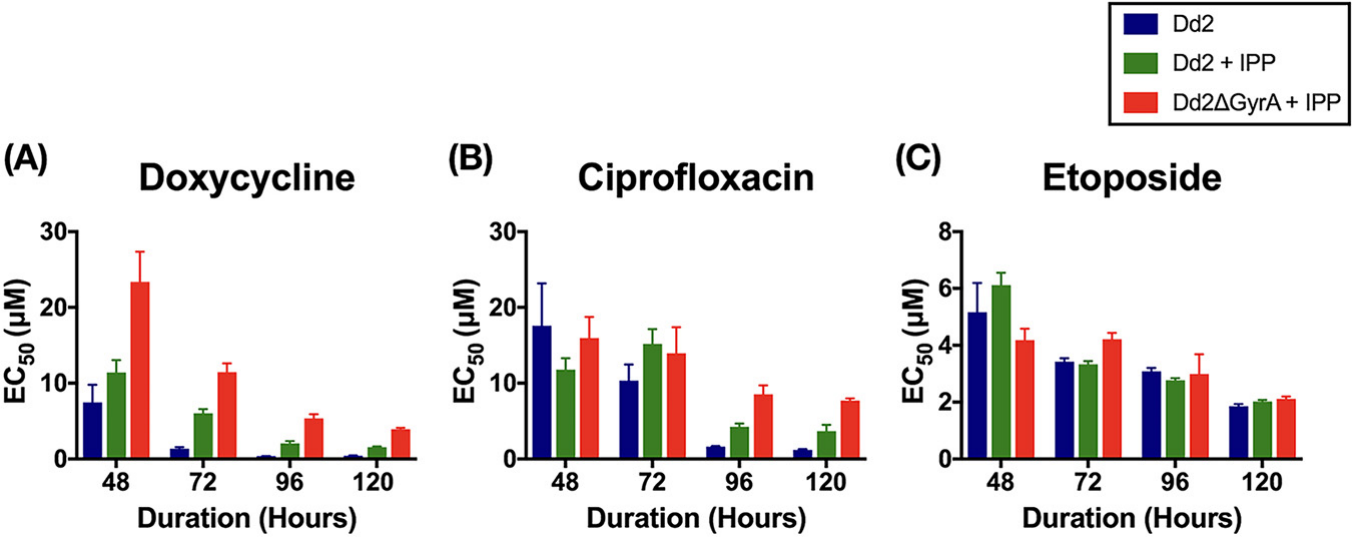
Drug sensitivity of different antibacterial compounds. Delayed-death phenotype was observed when Dd2 clone was treated with doxycycline or with ciprofloxacin (panels A and B, blue bars). The delayed inhibition at 96 h or 120 h could be rescued with IPP in both Dd2 and Dd2ΔGyrA clones (panels A and B, green and red bars). Inhibition by etoposide (control) showed no delayed death and no chemical rescue with IPP (C). Error bars represent the standard deviation of the results.

Previous studies have shown that parasites display a delayed-death phenotype when treated with antibacterials targeting apicoplast housekeeping processes (9, 34, 35). In those cases, parasites proliferated normally for one cycle but the daughter cells would lose their apicoplasts and could not complete another cycle. In order to determine the pharmacological susceptibilities of Dd2ΔGyrA grown with IPP, we applied doxycycline, ciprofloxacin, or etoposide for 48 h, 72 h, 96 h, or 120 h in drug-sensitivity assays. Doxycycline is an antibacterial known to inhibit bacterial protein synthesis, and ciprofloxacin is an antibacterial known to inhibit bacterial DNA gyrase. In both cases, inhibitory action on apicoplast was expected to cause a delayed-death phenotype in the parasite. On the other hand, etoposide is known to inhibit nuclear topoisomerase II and delayed-death phenotype was not expected. Indeed, Dd2 exhibited a delayed-death phenotype when treated with doxycycline or ciprofloxacin. When IPP was supplemented in the assay (Dd2+IPP), chemical rescue of apicoplast-specific inhibition was observed, consistent with previous findings involving other apicoplast targets (26, 36, 37). Dd2ΔGyrA supplemented with IPP displayed an inhibitor-response profile similar to that of a Dd2 clone (Fig. 6). This suggests that our CRISPR-generated Dd2ΔGyrA parasite line displays characteristics similar to those of the apicoplast-minus parasites generated through inhibition by small molecules. Inhibition by the control compound, etoposide, did not result in delayed death and did not allow chemical rescue with IPP. This indicated that our parasite line was not altered in regard to nuclear topoisomerase activity.

**FIG 7:**
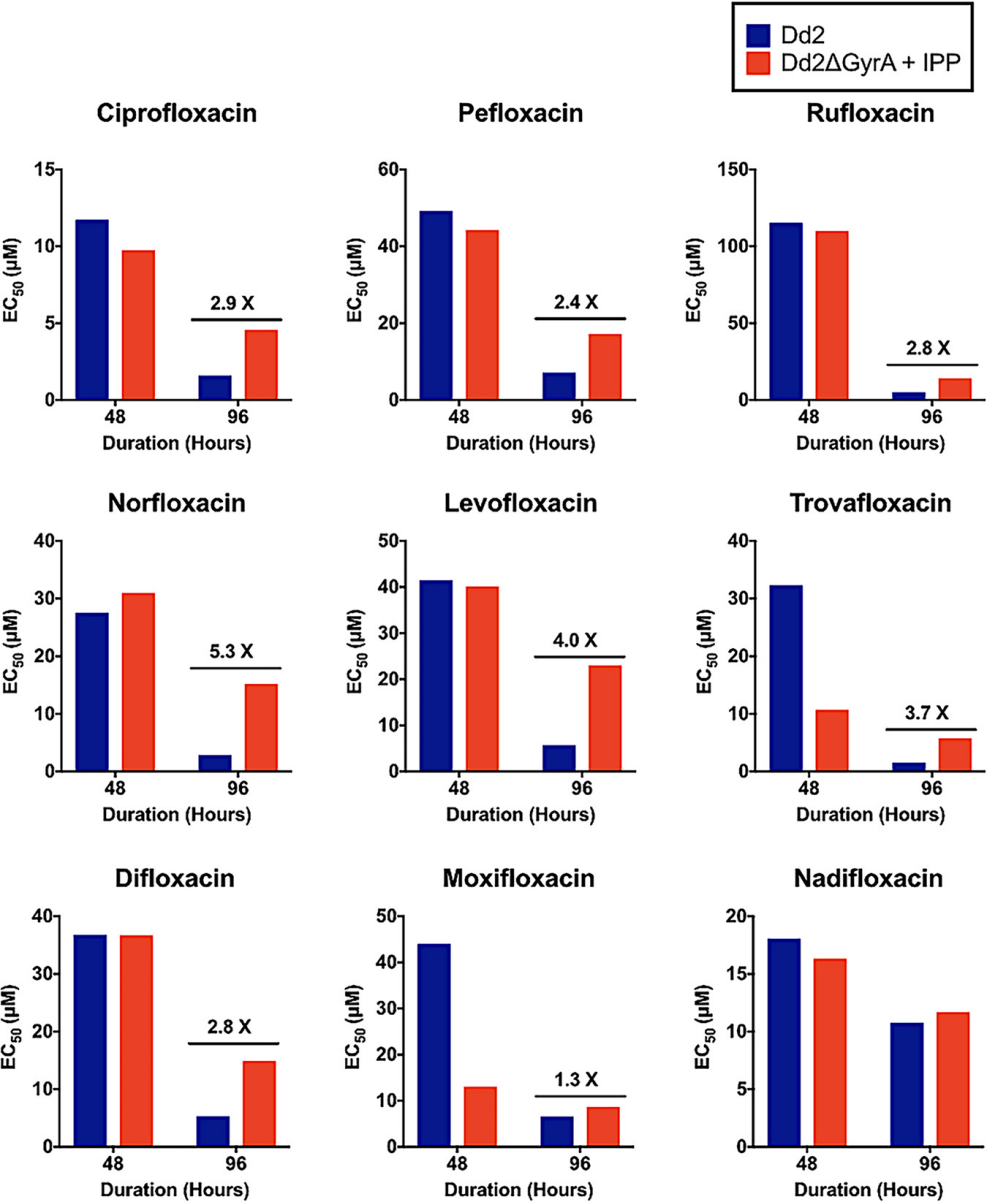
Drug sensitivity of fluoroquinolone derivatives. Dd2ΔGyrA clone exhibits similar inhibition and IPP-rescue profiles when tested with ciprofloxacin, pefloxacin, rufloxacin, norfloxacin, levofloxacin, trovafloxacin, and difloxacin. No significant delayed-death or IPP-rescue effects were seen in the Dd2ΔGyrA when tested with moxifloxacin or nadifloxacin. The bars were derived from EC_50_ curves at many different inhibitor concentrations.

In follow-up studies to ciprofloxacin, various other commercially available fluoroquinolone compounds were tested to determine their effect on the Dd2ΔGyrA clone (Fig. S1). The majority of the fluoroquinolone compounds displayed inhibition and IPP-rescue profile similar to those of ciprofloxacin. Two compounds were different: moxifloxacin and nadifloxacin did not show delayed death or IPP-rescue effect when tested on the Dd2ΔGyrA clone (Fig. 7). Other compounds such as flumequine, cinoxacin, and nalidixic acid displayed 50% effective concentration (EC_50_) values of >125 μM, and their full inhibition profiles were not determined (results not shown).

## DISCUSSION

In the present study, CRISPR/Cas9-mediated gene knockout of *Pf*GyrA confirms its importance to the apicoplast in blood-stage forms of malaria parasites. Immunofluorescence and whole-genome sequencing showed that disruption of the *PfgyrA* nuclear gene led to the loss of the apicoplast. This supports previous suspicions based on bioinformatics that *Pf*GyrA would be coded for in the nucleus and synthesized in the cytoplasm and that the protein product would be transported to the apicoplast, where it would have a major role in the maintenance of the integrity of the apicoplast (17).

The IPP chemical rescue strategy proved to be pivotal and necessary to allow for the potentially lethal disruption of the apicoplast-targeted DNA gyrase. Yeh and DeRisi first showed that IPP can be used to chemically rescue parasites that had been treated with apicoplast-targeting drugs (26). Similarly, Uddin et al. also demonstrated that a wide range of apicoplast-targeting drugs could be validated with the IPP chemical rescue method (37). For instance, for parasites that exhibit delayed-death phenotype when treated with doxycycline or ciprofloxacin, the inhibition could be chemically rescued with IPP supplementation (37). For the present work, it appears that the *Pf*GyrA is only needed in the apicoplast and the IPP-rescue approach was sufficient to rescue the *Pf*GyrA knockout parasites. Presumably, abrogation of an essential topoisomerase in the apicoplast (Δ*Pf*GyrA) would lead to inefficient DNA replication and inefficient transcription in the apicoplast, followed by loss of translation and loss of multiple gene products encoded by the apicoplast. Ultimately, this would lead to a parasite without a functional apicoplast (experimentally, as long as IPP was provided exogenously to keep the apicoplast-less parasites alive).

Although the apicoplast can be lost through chemical inhibition by small molecules, there are several advantages of generating an apicoplast-minus parasite using a genomic approach directed at one specific gene with just one predicted function. First, the clean genetic knockout of *Pf*GyrA offered an unambiguous comparison to characterize inhibitors like ciprofloxacin. Ciprofloxacin appears to have a single target at low concentrations (*Pf*GyrA, which could be rescued with IPP). At higher ciprofloxacin concentrations, nonapicoplast targets in P. falciparum begin to be important as judged by inability to rescue parasites with IPP. Second, drug-sensitivity assays of various fluoroquinolone compounds also revealed that there may be more than one mode of action of this class of compounds on malaria parasites, one with an apicoplast target (DNA gyrase) sensitive to low micromolar drug concentrations and the other with a nonapicoplast target that required a higher concentration of ciprofloxacin (Fig. 7). On the surface, while the ciprofloxacin analogs appear similar to the parent compound, they showed variations in potency and mode of parasite inhibition. In particular, nadifloxacin and moxifloxacin (both fluoroquinolone derivatives) did not exhibit a delayed-death phenotype and were not rescued by IPP. The 50% inhibitory concentration (IC_50_) values for this class of compounds against 3D7 parasites range from 4.5 to 142.9 μg/ml. These results suggest that, unlike many ciprofloxacin analogs that appear to primarily be *Pf*GyrA inhibitors and have delayed death, nadifloxacin and moxifloxacin are fast-acting antimalarials acting through a different mode of action. Overall, gene editing of *Pf*GyrA allows for such formal dissection and comparisons of antimalarial activity directed at this high-value target. In the future, as we reach for novel potent inhibitors that specifically target *Pf*GyrA from malaria parasites, the present validation model would allow us to stay on track.

## MATERIALS AND METHODS

### Plasmids construction.

Plasmids pDC2-gRNA-Cas9 and pDC2-donor-bsd were provided by Marcus Lee (Wellcome Sanger Institute, UK). Plasmid pDC2-gRNA-Cas9 was modified to include a 20-nucleotide gRNA sequence (ATAGGTAAATATCATCCACA) targeting *PfgyrA*. gRNA-oligonucleotide 1 (5′-ATTGATAGGTAAATATCATCCACA) and gRNA-oligonucleotide 2 (5′-AAACTGTGGATGATATTTACCTAT) were phosphorylated and annealed using T4 polynucleotide kinase in a thermocycler by incubating at 37°C for 30 min, increasing the heat to 94°C for 5 min, and then ramping down to 25°C at 5°C/min. Annealed gRNA-oligonucleotides were ligated with BbsI-digested plasmid. The sequencing primer (gRNA primer) was used for sequencing to confirm insertion of gRNA into the plasmid. To facilitate homology recombination, the plasmid pDC2-donor-bsd was modified to carry homology arms of *PfgyrA* (PlasmoDB ID PF3D7_1223300) flanking the selectable marker blasticidin-S deaminase (BSD). 5′ Homology arm of 307 bp starts from nucleotide +415 of the GyrA coding sequence, while 3′ homology arm of 306 bp starts from nucleotide +740. Nonsense mutations were inserted at nucleotides 437 to 438 (CT→GA) to introduce a stop codon at the beginning of the *PfgyrA* gene to stop further translation of the protein. 5′-GyrA-F and 5′-GyrA-R primers were used to amplify the 5′ homology arm from clone Dd2, GyrA-BSD-F and GyrA-BSD-R primers were used to amplify BSD selectable marker from the pDC2-donor-bsd plasmid, and 3′-GyrA-F and 3′-GyrA-R primers were used to amplify the 3′ homology arm from the Dd2 clone. These three PCR fragments were then assembled into a single piece that carried a BamHI site on the 5′ end and a SacI site on the 3′ end. The final assembled PCR construct was then digested with BamHI and SacI and ligated into the doubly digested pDC2-donor-bsd plasmid.

### Parasite cultures and transfection.

Parasites were cultivated using established methods with some changes (38, 39). Cultures of Plasmodium falciparum clone Dd2 were grown *in vitro* at 37°C in solutions of 2% hematocrit (serotype A positive human erythrocyte) in RPMI 1640 medium (Invitrogen) containing l-glutamine and 25 mM HEPES and supplemented with 0.5% Albumax I and 0.1 g/liter hypoxanthine (ThermoFisher). Cultures were maintained in sterile, sealed flasks and flushed with a blood gas mixture (5% CO_2_, 5% O_2_, and 90% N_2_). Transfections were done with the direct electroporation method on sorbitol-synchronized ring-stage parasites as described previously (31, 32). Briefly, Dd2 clone was electroporated with 100 μg of each plasmid using Bio-Rad Gene Pulser II set at 0.31 kV and 960 μF. Selection pressure (2.5 nM WR99210 and 2 μg/μl blasticidin) and 200 μM IPP (Isoprenoids, LC and home supplies) were applied 48 h posttransfection. Media supplemented with IPP and selection pressure were renewed every day for 6 days. On day 8 posttransfection, selection pressure was removed and medium was supplemented with IPP and changed every other day. Parasite proliferation was monitored by Giemsa-stained thin-smeared blood samples taken at each medium change three times a week.

### Synthesis of isopentenyl pyrophosphate.

Isopentenyl pyrophosphate was synthesized from 3-methyl-3-butene-1-ol according to the procedure of Davisson and coworkers depicted in Scheme 1 in the supplemental material (40). The synthesized IPP was first passed through cation exchange resin (ammonium form) for complete ion exchange. Subsequent purification on cellulose flash column chromatography resulted in a pure final product. Detailed synthesis procedures are outlined in the supplemental material.

### Analysis of Dd2ΔGyrA clone.

Parasite pellets from Dd2ΔGyrA were lysed using 0.15% saponin solution, and genomic DNA was extracted using Qiagen DNA minikit, according to the manufacturer’s instructions (41). Targeted gene disruption and BSD-cassette integration were verified by PCR using BIO-X-ACT short mix (Bioline LLC) with different primer sets as described in Results (see Fig. 2). PCR products were then treated with EXO-SAP-IT PCR cleanup reagent (ThermoFisher) and sent for sequencing using the same primers.

### Whole-genome sequencing analysis.

Genomic DNA of 400 ng from synchronized ring-stage Dd2ΔGyrA clone were sent to MedGenome, California for genomic libraries preparation and whole-genome sequencing. Paired-end reads were trimmed from bases with a quality score below 28 (Phred) using TrimGalore and then mapped to the 3D7 reference genome (Pf3D7v9) using BWA. Downstream processes were done following GATK (Genome Analysis Toolkit) best practices pipeline, where reads were sorted and duplicated reads were marked using Picard tools (42, 43). After Indel realignment and base recalibration steps using GATK software, final reads were compiled using SAMTools to generate variant calling files. Coverage of each chromosome including mitochondria and apicoplast was generated using SAMtools from the final pileup file, from which read depth at each nucleotide position was recorded. Results (see Fig. 4) were generated into coverage plots using Matplotlib.

### Drug-sensitivity assay.

Drug-sensitivity assays were performed in 96-well plates containing serial dilution of test compounds in triplicate. Medium was supplemented with 200 μM IPP as indicated. To determine the EC_50_ of different compounds, plates of 0.5% parasitemia were incubated for 48 h or 72 h. To determine if parasites clones show delayed-death phenotype, cultures were initiated at 0.2% parasitemia and incubated for 96 h or 120 h, and 75% of the medium was exchanged every 48 h. For all assays, parasitized cells were stained using SYBR green I (Invitrogen) and counted by flow cytometry (BD Accuri C6). Parasite proliferation in each well was expressed as a percentage of the solvent control. EC_50_ was determined using GraphPad PRISM software.

### Immunofluorescence microscopy.

Dd2 and Dd2ΔGyrA were incubated in 100 nM MitoTracker Red CMXRos (Invitrogen) stain for 30 min at 37°C. Cells were then prepared for microscopy as illustrated previously (44). Briefly, cells were washed and fixed in 4% paraformaldehyde (Electron Microscopy Sciences) with 0.0075% glutaraldehyde (Electron Microscopy Sciences) for 30 min at room temperature. Cells were then permeabilized with 0.1% Triton X-100 in PBS for 10 min and blocked with 3% bovine serum albumin/phosphate-buffered saline (BSA/PBS) for 2 h with end-over-end rotation. Cells were then incubated with 1:500 diluted ACP antibody for 1 h and then incubated with 1:500 diluted anti-rabbit secondary antibody conjugated with Alexa Fluor 488 for 30 min. Nuclear DNA was stained with 1 μg/ml DAPI for 10 min at room temperature. Cells were mounted onto slides with Hard Mount VectaShield (Vector Laboratories) and sealed. Fluorescence images were obtained on a Nikon Eclipse Ti-E equipped with a camera using a 100×/1.4 oil immersion objective. Images were analyzed using Nikon NIS-Elements software.
