# Supplementary material for: Properties of Plasmodium falciparum with a Deleted Apicoplast DNA Gyrase
Source: Antimicrob Agents Chemother. 2021 Aug 17;65(9):e00586-21. doi: 10.1128/AAC.00586-21 (PMC8370227; doi:10.1128/AAC.00586-21)
Supplement: Supplemental file 1 — Fig. S1 and Table S1. Download AAC.00586-21-s0001.pdf, PDF file, 0.2 MB [file aac.00586-21-s0001.pdf]

Supplementary Documents

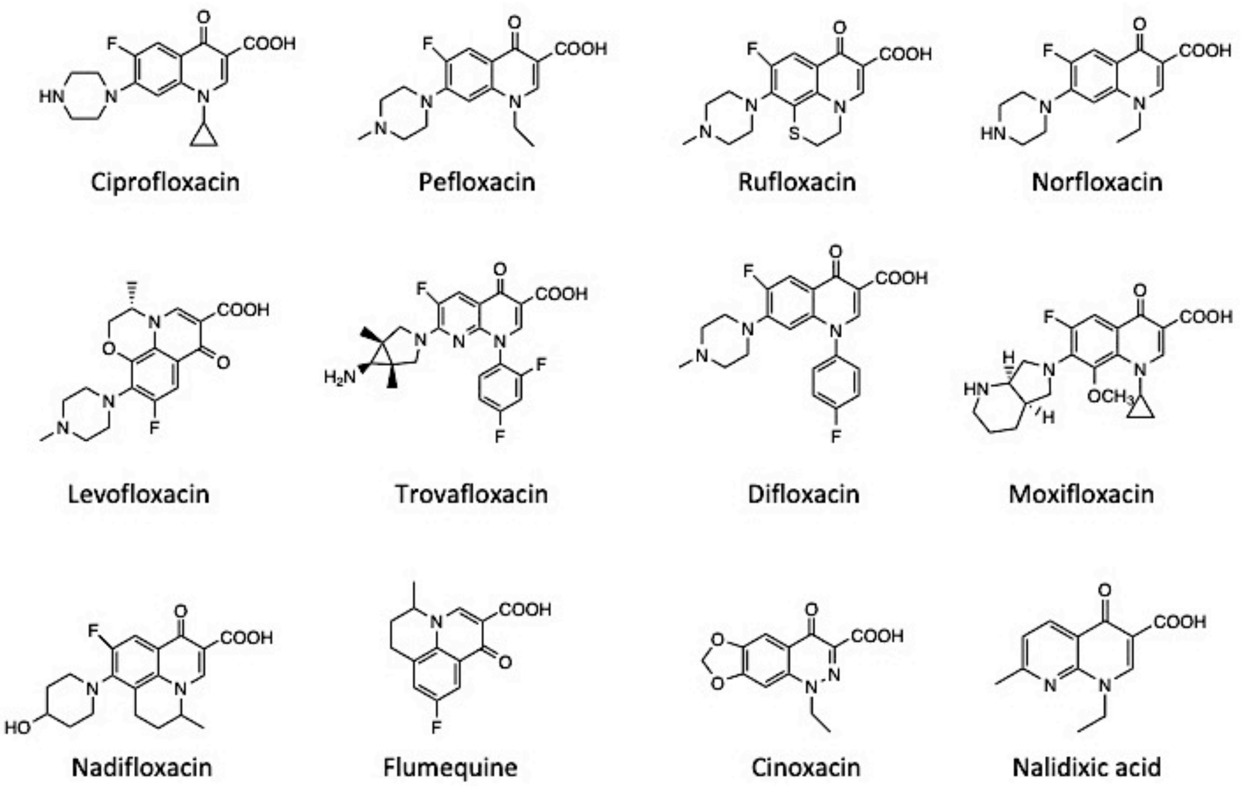

Figure S1. Structures of fluoroquinolone derivatives. Ciprofloxacin and pefloxacin were purchased from Sigma Aldrich and dissolved in water. Norfloxacin, levofloxacin, trovafloxacin, difloxacin, nadifloxacin, flumequine, cinoxacin, and nalidixic acid were purchased from Sigma Aldrich and dissolved in DMSO. Rufloxacin was purchased from MedChem Express and dissolved in water while moxifloxacin was purchased from Cayman Chemicals and dissolved in DMSO.

Table S1. List of primers used in this study.

Primer	Sequence (5'->3')
gRNA primer	GTTGTGTGGAATTGTGAGCGG
5' GyrA –F	TTACTCGGATCCAAAGATGATATAGTTAATGCATCTAATGATATAACAAATGA
5' GyrA – R	AAGCGAATTAGCTAAGCATGCTACCTATAACTTCACCAACTAT
GyrA-BSD-F	ATAGTTGGTGAAGTTATAGGTAGCATGCTTAGCTAATTCGCTT
GyrA-BSD-R	CTAAAGCATCATATACACTCTTATTTTCTCTGCGGTTTAATAAATATG
3' GyrA –F	CATATTTATTAAACCGCAGAGAAAATAAGAGTGTATATGATGCTTTAG
3' GyrA –R	TGATAAGAGCTCAAGGTATACTGCTTAATATACTTAC
PCR – P1	GAGTAATCGTTCAACATATACAG
PCR – P2	ACCAGTTGAAAAATCAGGTCC
PCR – P3	GTGGATTCTTCTTGAGACAAAG
PCR – P4	TCAAAGCCATAGTGAAGGAC

Synthesis of Isopentenyl Pyrophosphate

Isopentenyl pyrophosphate (IPP) was synthesized from the corresponding alcohol (**1**) according to the procedure of Davisson and co-workers as depicted in scheme 1[40]. 3-methyl-3-butene-1-ol (**1**) was converted to 3-methylbut-3-en-1-yl 4-methylbenzenesulfonate using para toluenesulfonyl chloride in dichloromethane. Further, the tosyl intermediate (**2**) was treated with tris(tetra-n-butylammonium) hydrogen pyrophosphate to obtain the Isopentenyl pyrophosphate (IPP). The final product was first passed through cation exchange resin (ammonium form) for complete ion-exchange. Subsequent purification on cellulose flash column chromatography yielded the final product (IPP).

Scheme 1:

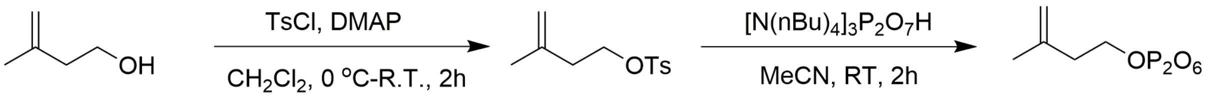

1 **2** **IPP**

3-methylbut-3-en-1-yl 4-methylbenzenesulfonate (**2**):

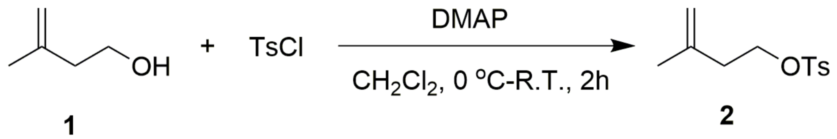

P-Toulenesulfonyl chloride (2.2 g, 11.6 mmol) and 4-(N, N-dimethylamino)pyridine (1.5 g, 12.76 mmol) were mixed with 50 ml of anhydrous dichloromethane in a 100 ml round bottom flask at 0 °C, under argon. A solution of 3-methyl-3-butene-1-ol (1) (1 g, 11.6 mmol) in anhydrous dichloromethane (5 ml) was then slowly introduced with a syringe through a septum in the flask, and the ice bath was then removed. The reaction was stirred for 1-2 h until silica-gel TLC (Hexane/Ethylacetate, 85:15 (v/v) indicated that reaction was complete. The reaction mixture was quenched with water (10 ml) at 0 °C and poured into 1 M HCl. The layers were separated and the organic extract was washed with water (20 ml), concentrated in vacuo, and purified by flash column chromatography. Using Hexane/Ethylacetate (5:1) as the eluent, the concentrated title compound 3-methylbut-3-en-1-yl 4-methylbenzenesulfonate (2) appeared as a color less oil (2.31 g, 80% yield). ^{1}H NMR ($CDCl_3$) δ: 1.67 (s, 3H), 2.36 (t, *J* = 6.8 Hz, 2H), 2.48 (s, 3H), 4.15 (t, *J* = 6.8 Hz, 2H), 4.69 (brs, 1H), 4.80 (brs, 1H), 7.36 (d, *J* = 8.0 Hz, 2H), 7.80 (d, *J* = 8.0 Hz, 2H). ESI-MS m/z: 258 $[M+NH_4]^+$.

3-Methyl-3-butenyl diphosphate (Isopentenyl pyrophosphate, IPP) (3):

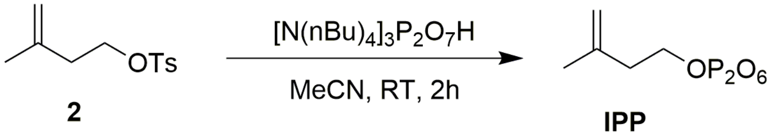

To 3-methyl-3-buten-l-yl tosylate (**2**) (288 mg, 1.20 mmol) was added tris(tetra-n-butylammonium) hydrogen pyrophosphate (3.25 g, 3.6 mmol) in 3.5 mL of acetonitrile, and the resulting solution was allowed to stir for 2 h. The resulting material was converted to the ammonium form with 30 equiv of DOWEX AG 50W-X8 cation exchange resin (100-200 mesh). After lyophilization, the resulting powder was dissolved in 3 mL of 0.1 M ammonium bicarbonate, extracted twice with 7 mL of 1:l (v/v) acetonitrile/isopropyl alcohol. Flash chromatography on a 3.5 cm X 15 cm cellulose column (4.5:2.5:3 (v/v/v) isopropyl alcohol/acetonitrile/0.l M ammonium bicarbonate yielded 59 mg (20% yield) of **IPP** as a white solid; ^{1}H NMR (300 MHz, D_2O) δ: 1.67 (s, 3H), 2.29 (t, 2H, *J* = 6.3 Hz), 3.96 (qd, *J* = 6.6, 2.3 Hz, 2H), 4.70 – 4.85 (m, 2H); ^{31}P NMR (32 MHz, D_2O) δ: -10.41 (d, *J* = 20.1 Hz, 1P), -7.54 (d, *J* = 20.1 Hz, 1P). ESI-MS m/z: 247.4 $[M+H]^+$.
